# Odevixibat Treatment of Alagille Syndrome: A Case Report

**DOI:** 10.1097/PG9.0000000000000301

**Published:** 2023-03-24

**Authors:** Rainer Ganschow, Christof Maucksch

**Affiliations:** From the * Department of Pediatrics, University Children’s Hospital, Bonn, Germany; † Albireo Pharma, Inc., Boston, MA.

**Keywords:** bile acids and salts, cholestasis, pruritus

## Abstract

A male pediatric patient with elevated liver enzyme and bile acid levels, bile duct hypoplasia, mild liver fibrosis, and pruritus was initially diagnosed with progressive familial intrahepatic cholestasis. The patient did not respond to treatments of ursodeoxycholic acid and naltrexone. Subsequent treatment with odevixibat resulted in improvements in serum bile acid levels and pruritus within a few weeks of initiation. During the course of odevixibat treatment, genetic testing results and additional clinical findings indicated a diagnosis of Alagille syndrome, a condition that shares some clinical features with progressive familial intrahepatic cholestasis. Odevixibat treatment was continued off label, during which time the patient’s serum bile acid levels dropped to within the normal limit and pruritus was completely ameliorated. This report suggests odevixibat may be an effective treatment option for Alagille syndrome.

## INTRODUCTION

Alagille syndrome (ALGS), caused by mutations in *JAG1* or *NOTCH2*, has variable presentation and may affect multiple organs, which can make diagnosis challenging (1). Diagnostic criteria include cholestasis, cardiovascular defects, ocular posterior embryotoxon, atypical facial features, and/or skeletal malformation (1). Patients with ALGS often suffer from impaired health-related quality of life (QoL) and persistent cholestasis (2). Cholestasis results in elevated serum bile acid levels (sBAs) and abnormal liver enzyme levels, as well as symptoms like growth delay, xanthoma development, and severe pruritus, which can be particularly burdensome (1,2). Liver disease in ALGS can be progressive and may ultimately require liver transplantation (1).

The pathophysiology of ALGS is characterized by bile duct paucity, which can result in accumulation of bile acids in the liver and subsequent spillover into circulation (1,3). While not fully understood, elevated sBAs have been proposed as a possible factor contributing to cholestatic pruritus (3). ALGS has been managed with supportive medical therapies like choleretic agents (eg, ursodeoxycholic acid [UDCA]), other medications (eg, rifampin), or surgical procedures like biliary diversion (1).

The ileal bile acid transporter (IBAT) is a regulator of the enterohepatic bile acid circulation (3). Given the central feature of cholestasis in ALGS and the role of IBAT in bile acid homeostasis, IBAT inhibitors may help alleviate symptoms of ALGS. In the phase 2 ICONIC trial (Long-term, Open-label Study With a Double-bl**i**nd, Placebo-controlled, Randomized Drug Withdrawal Period of LUM001, an Api**c**al S**o**dium-Depe**n**dent B**i**le A**c**id Transporter Inhibitor, in Patients With Alagille Syndrome), the IBAT inhibitor maralixibat significantly improved sBAs, pruritus, and other symptoms in patients with ALGS (4). Maralixibat is now approved in the United States for treatment of cholestatic pruritus in patients ≥1 year old with ALGS (5). The IBAT inhibitor odevixibat is indicated for treatment of pruritus in patients ≥3 months old with progressive familial intrahepatic cholestasis (PFIC), another pediatric cholestatic liver disease, in the United States and for treatment of PFIC in patients ≥6 months old in the European Union (6,7). Odevixibat has also been evaluated in 6 patients with ALGS in a phase 2 open-label study over 4 weeks; in that study, odevixibat reduced sBAs, pruritus, and sleep disturbance in a majority of those patients (8). Odevixibat may warrant further investigation as a possible therapeutic option for ALGS. We describe the effects of odevixibat treatment over 8 months in a pediatric patient with genetic confirmation of ALGS who had highly elevated sBAs with severe pruritus.

## CASE REPORT

A male infant, born in August 2017, presented with prolonged jaundice after birth. Evaluation revealed hyperbilirubinemia, elevated liver enzyme levels (ie, alanine aminotransferase, aspartate aminotransferase, and gamma-glutamyl transferase), and a normal total cholesterol level (105 mg/dL). The patient was referred to specialist care at 2 months of age. Semiannual ultrasound studies indicated splenomegaly and mild-to-moderate signs of portal hypertension. The patient had normal facial features, no skeletal abnormalities, and no overt cardiac symptoms. Initial diagnosis of PFIC was established at 7 months of age. Genetic confirmation was not pursued at the time, given that the patient’s clinical features were consistent with PFIC, and no effective treatment for familial cholestasis was available at the time. In addition, genetic testing was inaccessible in the surrounding area during this period. At 1 year of age, a liver biopsy suggested bile duct hypoplasia and mild fibrosis without additional details due to the very small tissue sample available for analysis.

Severe pruritus began at age 2 years. The patient’s mother observed chronic scratching of the ears, arms, legs, and feet that resulted in bleeding, disturbed the child’s sleep, and made it difficult for him to focus during the day. The child’s pruritus also had a negative impact on the family’s QoL. Additionally, the patient had persistent abdominal pain and painful, irregular bowel movements. At age 3 years, the patient could only attend kindergarten for 4 hours per day due to symptoms.

**FIGURE 1.**
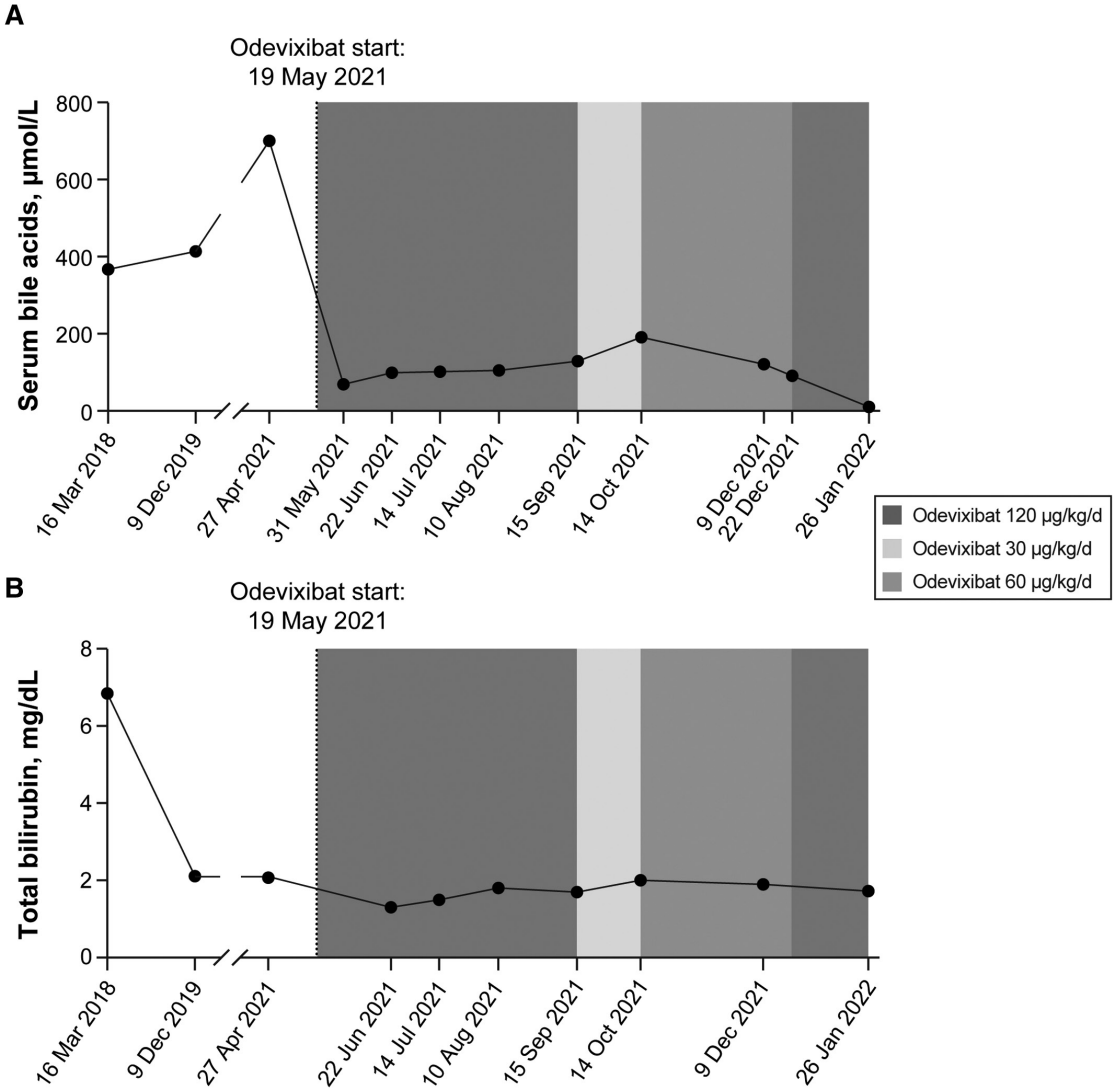
Serum bile acid (A) and total bilirubin (B) levels before and after odevixibat treatment in the patient found to have Alagille syndrome. Vertical dashed lines indicate odevixibat start, and shading indicates the different doses of odevixibat that were administered.

During this clinical course, the patient received UDCA (15 mg/kg body weight/d) and naltrexone (1 mg/kg/d). No significant clinical improvement was observed with these treatments, and the patient consistently had elevated sBAs (Fig. 1A), serum bilirubin (Fig. 1B), serum liver enzyme levels, and ongoing pruritus. In April 2021, a compassionate-use program for odevixibat in patients with PFIC became available in Germany. Given the patient’s pre-established clinical diagnosis and lack of response to other medical therapies, the patient started odevixibat 120 μg/kg/d as part of this program on May 19, 2021. The patient’s sBAs (Fig. 1A) and serum total bilirubin (Fig. 1B) quickly decreased. The approximately 90% drop in sBAs was particularly dramatic, going from 700 μmol/L on April 27, 2021, to 69 μmol/L on May 31, 2021. Alanine aminotransferase, aspartate aminotransferase, and gamma-glutamyl transferase levels rose in the months following odevixibat initiation (from 111 to 188 U/L, 116 to 140 U/L, and 187 to 487 U/L, respectively).

In August 2021, genetic testing was performed, and a heterozygous *JAG1* mutation (c.2917-10A>G) was found. This variant has been previously reported by Stalke et al (9) as a variant of unknown significance in a patient with a characteristic ALGS phenotype. In the current report, genetic testing of the patient’s parents found no evidence of the c.2917-10A>G variant in either the mother or the father, which suggests that the variant was a pathogenic de novo variant causing ALGS. Further diagnostic tests were undertaken at this time, and mild peripheral stenosis of the pulmonary artery and posterior embryotoxon were identified. The previous findings by Stalke et al (9), together with the patient findings described here (ie, genetic testing results and additional clinical findings), strongly suggest that the heterozygous c.2917-10A>G mutation is indeed pathogenic. Subsequently, the diagnosis of our patient was corrected to ALGS, and the patient became ineligible for the compassionate-use program in PFIC. Odevixibat treatment was then continued off label. Additional testing in November 2021 revealed an elevated total cholesterol level (292 mg/dL), which gave further support to the revised diagnosis of ALGS in this patient.

To fulfill potential payor requirements, the patient’s odevixibat dosage was changed multiple times, as follows: to 30 µg/kg/d from September to October 2021, which was associated with an increase in sBAs, to 60 µg/kg/d from November to mid-December, and back to 120 µg/kg/d in mid-December, which corresponded with a decline in sBAs (Fig. 1A). sBAs reached the normal range after 8 months of treatment (Fig. 1A).

**FIGURE 2.**
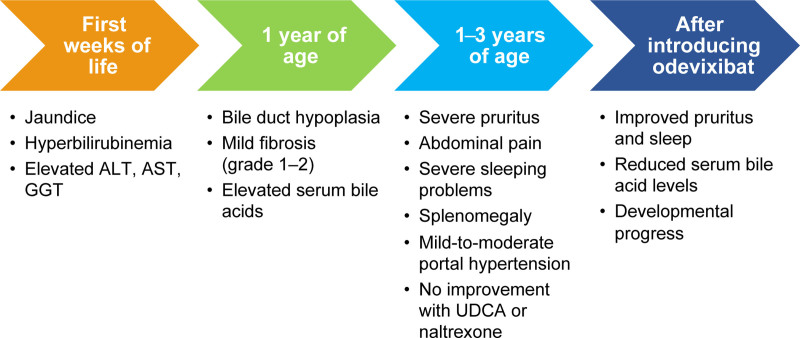
Flow diagram of key disease features throughout the life of the patient found to have Alagille syndrome and the impact of odevixibat treatment. ALT = alanine aminotransferase; AST = aspartate aminotransferase; GGT = gamma-glutamyl transferase; UDCA = ursodeoxycholic acid.

The mother reported that the patient started sleeping through the night within 4 days of starting odevixibat. Pruritus almost entirely disappeared within 2 weeks and stopped completely after 8 months. Furthermore, the patient’s bowel movements improved after 1 week, and he no longer suffered from abdominal pain. The patient was able to start going to school, with improved focus, for at least 6 hours per day after starting odevixibat. Both the parents and daycare staff noted significant developmental progress. QoL was reported to significantly improve for the whole family. A flowchart of key milestones of disease progression and treatment success is shown in Figure 2. The patient did not experience any adverse events with odevixibat.

## DISCUSSION

In addition to supporting findings from the phase 2 trial of odevixibat in 6 patients with ALGS (8), this case report provides the first evidence of sustained treatment benefits and real-world effectiveness with odevixibat in ALGS. This case report also highlights the high burden of disease in patients with ALGS. Pruritus was one of the most bothersome symptoms of ALGS that greatly affected the patient’s and his family’s daily life. As exemplified by this patient, off-label medications that have been traditionally used to treat pruritus may not always work in children with ALGS. Neither UDCA nor naltrexone successfully treated symptoms in this case. The IBAT inhibitor odevixibat was the first treatment that worked for this patient, with elimination of pruritus and rapid and robust reductions in sBAs. Importantly, odevixibat treatment also improved QoL for the patient and his family. Odevixibat was well tolerated in our patient, and treatment was associated with improved abdominal pain. This case emphasizes the need for additional medications for patients with ALGS with severe pruritus. Based on the success here, as well as data from a previous study of odevixibat in children with ALGS (8), odevixibat may be one possible alternative.

## ACKNOWLEDGMENTS

Prior to initiating any treatment, the treating physician received informed consent from the patient’s parents, and this, as well as the expanded access program protocol, were submitted to and approved by an Independent Ethics Committee. In addition, informed consent was obtained from the parents of the patient for publication of the case details presented here. We thank the patient’s family, who contributed to this case report.
